# Supplementary material for: SyNDI: synchronous network data integration framework
Source: BMC Bioinformatics. 2018 Nov 6;19:403. doi: 10.1186/s12859-018-2426-5 (PMC6219086; doi:10.1186/s12859-018-2426-5)
Supplement: Supplementary file 5 — Files of the example presented in Additional file 4. This additional file is a zip package that contains all files used in Additional file 4. Detailed descriptions of each of these files are presented in a README file included in this zip package. (GZ 30411 kb) [file 12859_2018_2426_MOESM5_ESM.gz › SyNDITools/Meme2Fimo/meme_4.9.0_4.tar.gz!/meme_4.9.0/doc/transfac2meme.html]

transfac2meme


# transfac2meme

**Usage:** `transfac2meme [options] <matrix file>`

**Description:**

Extract motifs from a TRANSFAC 'matrix.dat' file and print them in
MEME Version 4 format.

Counts are converted to frequencies using pseudo-counts determined by
the backgoround frequencies and the total pseudocounts (see -bg and
-pseudo, below). The log-odds matrix uses the background frequencies
in the denominator and is log base 2.

N.B. Dollar signs in TRANSFAC IDs are converted to underscores.

**Input:**

A file in TRANSFAC matrix format

**Options:**

-numbers
:   use numbers instead of strings as motif names

-use\_acc
:   use accession names instead of IDs

-ids <idfile>
:   keep any motifs listed in the file

-species <name>
:   keep only motifs for this species

-skip <ID>
:   skip this ID (may be repeated)

-bg <background file>
:   file with background frequencies of letters;default: uniform background

-pseudo <total pseudocounts>
:   add <total pseudocounts> times letter background to each frequency; default: 0

-logodds
:   print log-odds matrix, too; default: print frequency matrix only

-url <website>
:   website for the motif; The ID (or accession) is substituted for MOTIF\_NAME; default: no url

**Output**

Writes MEME format to standard output.

**Sample Input**

TRANSFAC file format
